# Realistic simulations of local field potentials in a slice

**DOI:** 10.1186/1471-2202-14-S1-P417

**Published:** 2013-07-08

**Authors:** Hanuma C Chintaluri, Helena Głąbska, Torbjørn Bækø Ness, Gaute T Einevoll, Daniel K Wójcik

**Affiliations:** 1 Department of Neurophysiology, Nencki Institute of Experimental Biology, Warszawa, 02-093, Poland; 2 Department of Mathematical Sciences and Technology, Norwegian University of Life Sciences, 1432 Aas, Norway

## 

The two key elements of realistic simulation of LFP are plausible model of neural activity, and the physical properties of the setup, tissue, and electrodes. To reduce the computational burden yet obtain a trustful rendering of LFP in specific experimental context different strategies have been tried. For instance, using total synaptic activity from a population of spiking neurons as a proxy for LFP, or hybrid strategies with network simulations of spiking neurons followed by computation of contributions to LFP from single cells driven by pre-calculated network dynamics. Tests of those strategies are impossible without ground-truth data including all the necessary components.

**Figure 1:**
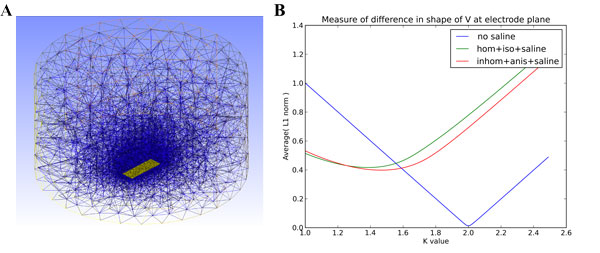
**A. The mesh in the Petri dish with saline (cylinder), slice is the yellow bar**. **B**. Average L1 norm of difference |k*V_hom _- V| as a function of k where V is the potential on the electrode plane in the three cases i)-iii).

Working towards a framework providing ground truth data for validating methods of analysis of extracellular potentials in cortical slices, **we investigated how the use of increasingly detailed physical models of slice tissue affects the resulting model LFPs**. Traub's model of thalamo-cortical loop [1] was simulated in NEURON and the extracellular potentials in uniform, homogeneous medium (V_hom_) were computed post-hoc from tracked trans-membrane currents. To verify the need for inclusion of experimental setup context we modeled the field in the tissue and saline using finite-element approach (FEM) with FENICS package with mesh generated with gmsh. The slice (yellow parallelogram) was placed in a saline solution (Figure 1A) and the cortical column was put in the middle of the slice, and we computed the extracellular potentials it generated at the electrode plane under the slice.

We modeled 3 different conductivity profiles within the slice and the saline:

**i) **'no saline' case: slice and saline with the same conductivity of 0.3 mS/cm

**ii) **'hom+iso+saline': homogeneous and isotropic slice of conductivity 0.3 mS/cm, saline: 3.0 mS/cm

**iii) **'inhom+anis+saline': inhomogeneous and anisotropic slice with conductivity profiles for the layers taken as mean values from [2].

We see that the inclusion of slice setup noticeably modifies the observed activity as both the amplitude and shape of the potential profile is changed (Figure 1B). However, inclusion of inhomogeneity and anisotropy in the computations does not lead to substantial changes of the profile. Indeed, inaccurate estimation of conductivity (see variability of results in [2]) will in general introduce bigger errors than in assuming homogeneous and isotropic tissue.
